# Hospital and Institutional News

**Published:** 1914-08-01

**Authors:** 


### HOSPITAL AND INSTITUTIONAL NEWS.

### THE WATER FAMINE AT WOLVERHAMPTON.

We have had to hold over till this week an account of the extraordinary water famine which has caused so much inconvenience and trouble at the hospital of that town. Due not to scarcity of water but to the breakdown of one of the Corporation's engines, the pumps would not raise enough water to give the necessary supply, and while the inconvenience in the houses of the districts may be easily imagined, at the hospital the supply became non-existent during the progress of operations in the theatre, and on one occasion was suspended for nearly ten hours on end. While the operations were in progress water had to be carried in odd buckets and jugs, and for a whole month extreme inconvenience has been caused. There have been two hundred patients in the institution, and children have had to be refused admission, and the question of closing wards has had to be considered. In view of this lengthy period of interruption considerable evidence will be required to substantiate to public satisfaction the statements of the Water Committee that everything possible has been done without delay. Apparently new apparatus has had to be awaited, but this seems to show either that due repairs have been neglected in the past, or that sufficient means to cope with breakdowns are not available on the spot, in either case an unsatisfactory explanation. Public institutions meanwhile have been urged to store all the water that they can.

### COMPLAINTS AGAINST A WANDSWORTH INFIRMARY.

A series of serious complaints have been made by the Mayor of Battersea with reference to the treatment of the patients in St. James's Infirmary, Wandsworth. The Mayor has made personal inquiries into the allegations, and the Guardians have referred them to the responsible committee for investigation. The mother of a boy patient was so indignant at the treatment her son is said to have received that, despite the fact that she was informed that it was dangerous to remove him, she at once did so. It was alleged that he had been allowed to lay on the verandah unprotected against the sun for a considerable time until his face was badly scorched, and that his food was served cold and in an unsatisfactory condition. It was stated that a sister and four nurses had charge of seventy-five patients, and consequently were unable fully to attend to the wants of all those in their charge. The allegations were of so grave a character that it is essential that a full inquiry should be held at once with a view to removing any causes of faulty administration and so restore the confidence of the poor in the infirmary. The Mayor of Battersea's evidence in support of his allegations is to be placed before the committee, who should come to a speedy decision on the matter.

### OVER THIRTY YEARS SECRETARY AND SUPERINTENDENT.

The sympathy of all hospital workers will be extended to Mr. Arthur Hulme, late Secretary and General Superintendent of the Queen's Hospital, Birmingham. Owing to ill-health, Mr. Hulme has had to resign the appointments which he has held for upwards of thirty years. During his period of office important reconstructions and enlargements have been carried out, the plans of which were published on pages 497-8 of The Hospital of August 12, 1911. In The Hospital of October 30, 1909, page 131, it is recorded that "at the Queen's Hospital the Uniform System of Hospital Accounts was born, and here also most of the principles of the plan which formed the basis upon which the present system of training nurses rests were first put into action." We have hoped for many years and shall continue to hope that the older portions of this hospital, constituting its surgical wing, will be rebuilt at the first opportunity. Mr. Hulme's field of work was deeply interesting. In accepting his resignation with great regret, the committee placed on record the expression of their high appreciation of Mr. Hulme's services to the hospital for over thirty years. We hope Mr. Arthur Hulme has retired on a pension, and that the pension will be found to be adequate. It would be interesting to have some information on this point.

### NEW SECRETARY OF THE LONDON LOCK HOSPITAL.

Mr. H. J. Eason, secretary of the Manchester Children's Hospital, Pendlebury, has been appointed secretary of the London Lock Hospital, in Harrow Road. His many friends in Manchester and elsewhere will congratulate him and the hospital on this appointment, for he has been anxious for some time to leave the North for London, and the Children's Hospital, Pendlebury, has been known as a model of what a children's hospital ought to be to all interested in children's hospitals. The Lock Hospital may be congratulated, therefore, on the new secretary, who succeeds Mr. Edwin Eddison; but it is not only Pendlebury which will miss Mr. Eason, for, among other things, he has been an active member of the Association of northern secretaries, a useful if unobtrusive body, the members of which visit each other's institutions and discuss administrative points in the privacy secured by the absence from their body of all junior officials. The number of beds at Pendlebury is 180, and that at the Lock Hospital 145, with serious extra duties for its Secretary. Mr. Eason's ambition has been to take up work in London. His appointment is also of importance, since there is great scope at the London Lock Hospital for a keen organiser, who will bring the institution's administration up to date in all respects, and Mr. Eason has shown a talent in this respect which promises well for the success of his new work.

### THE VALUE OF THE UNIFORM SYSTEM.

The individual secretaries of the separate institutions do not always appreciate the trouble involved in keeping their accounts on the Uniform System, and any reluctance on their part is increased if the accounts have previously been kept in any other manner. Some only pretend to employ the Uniform System, but do not actually put its instructions into practice; yet how useful that system is may be seen from the attempt made at Macclesfield General Infirmary to study economy and comparative methods of securing it. The Special Committee which has been appointed to inquire into the finances of the institution, instructed the secretary, Mr. A. E. Hanrahan, to get particulars of the average stay of each patient in institutions of similar size, the amount of provisions received in a week, and the list of meals; and, thirdly, the cost of maintenance per bed under the heads of surgery and dispensary, nursing staff, domestic staff, and maintenance. Now it is obvious to anyone that these particulars are made possible only by uniformity in the account-keeping of the institutions consulted, and the inquiry here instituted should bring home to committees who have not yet adopted the system the paramount importance of embracing it, not merely because the system is of value in itself, but also because, as this case illustrates, mutual aid between institutions on financial matters depends upon its adoption throughout the entire hospital world.

### THE MEDICAL SERVICES OF BRADFORD.

A big scheme, involving an expenditure of £40,000, has been put forward by the Health Committee of Bradford for the centralisation of the medical services. They proposed that the tuberculosis dispensary, the school clinic, and the bacteriological laboratory should be housed in new offices at Horton Lane. Councillor E. J. Smith, chairman of the Health Committee, declared that these departments should be co-ordinated, and that the top floor of the Town Hall, where the department's offices are now, caused grave risk to serious cases attending there. Councillor James Hill, while opposing the project, preferred the old infirmary site. In reply to this Mr. Smith said that it would be a very unfair thing to make the Health Department "the victim of the transaction" by which the Council acquire the site of the Royal Infirmary for £100,000. The proposal was finally rejected by a two-thirds majority, and the general feeling of those who voted against it appears to be that it would be only a return to the old plan of scattering the city's public departments all over the town, which was abandoned some years ago, when the Town Hall was extended. As, however, the accommodation even in the extended Town Hall is being severely strained, with the result that certain departments will, after all, have to seek new quarters before long, there is surely something to be said for endeavouring to find a new site where those which can no longer be accommodated in the Town Hall may be grouped together. Whether the infirmary site would serve this purpose is a matter for local decision, but we understand that it was intended for public use when the purchase was under discussion.

### GUARDIANS AND THE MENTALLY DEFICIENT.

Mr. R. Woolley-Walden, chairman of the Metropolitan Asylums Board and the City of Westminster Union, presided last week at a special meeting of London Boards of Guardians further to discuss Sir W. Byrne's recent paper on "The Administration in London of the recent Mental Deficiency Act." The Asylums Board, he said, thought that so long as the two bodies continued to deal with defectives the existing division of classes between them should be maintained, and that accommodation for new classes now dealt with by the Board should continue to be provided by the Board in agreement with the County Council. A conference between the two was being arranged, and the Asylums Board hoped the Metropolitan Guardians would consider whether by an arrangement come to between themselves they could place any of the existing Poor-Law accommodation at the Board's disposal, so as to avoid unnecessary rebuilding. Sir W. Byrne, after replying to numerous questions, mentioned the Board of Control's suggestion that a voluntary local association, comprising persons like Guardians, the National Society for the Prevention of Cruelty to Children, and other suitable people, should be formed in every area, on a representative basis, to undertake such duties as the local authorities might think within their scope. Local societies, he added, had already been started or had coalesced in many places, and a sum of money was available for distribution among them as the work developed. It is hoped officially that by means of the voluntary element existing hostility to the Act will be much reduced.

### EARLY MENTAL CASES IN MELBOURNE.

Dr. W. E. Jones, Inspector-General of the Insane (Victoria), who is a keen student of preventive methods, recently made a proposal to the committee of the Melbourne General Hospital that psychiatric wards for the observation of mental disease in the earliest stages might be established therein. He naturally appreciates the fact that insanity usually commences long before it would be possible to "certify" that the person is insane, and thus the most valuable period for treatment is lost. The committee and its honorary medical staff replied that the staff strongly supported the principle, but could not at present recommend that psychiatric wards should be established at the Melbourne Hospital. The Melbourne Hospital should assist, states the report, by the establishment of a special out-patient department, under a specialist, and by the admission into one or other of the present wards of suitable cases, upon order from a member of the honorary medical staff or superintendent. In the opinion of the staff, the best way of treating early mental cases generally would be to throw open the Acute Mental Hospital, and all licensed private houses, to all such cases without any preliminary certification, and to provide the Acute Mental Hospital with a thoroughly efficient out-patient department. In view of Dr. Armstrong Jones's recent proposals the above report is of special interest. Dr. Jones was much disappointed at this reply, and is confident that a hospital committee of a less reactionary spirit will be found to undertake the work.

### A SUBSCRIBER'S ENTHUSIASM AT BRISTOL.

A sun balcony has been built and opened at the Bristol Orthopædic Hospital and Home for Crippled Children at Redland. It is the result of a successful appeal which was issued by Mr. J. H. Howell, J.P., an old subscriber to the institution. On his first visit to the institution he was so much impressed by the value of the work performed that he fell in with the suggestion of Mr. H. Chitty, the honorary surgeon, that an effort should be made to secure open-air accommodation for the tuberculous children. The new balcony which has resulted adjoins the main building, and possesses blinds which can be drawn to protect the patients in rough weather. Measuring 30 feet by 10 feet, and providing sleeping accommodation, the new balcony, with certain improvements that have accompanied it, has cost some £200. New bathrooms and a new heating apparatus are also in hand. It is a remarkable tribute to Mr. Howell's energy and enthusiasm that the necessary money was subscribed within a week after the issue of the appeal, so that Mr. William Henderson, the honorary treasurer, was able to give the order almost at once. This shows how much can be done if energy is thrown into the work, and how advisable it is for subscribers to enter personally into the life and the needs of the hospitals that they support.

### PAY-WARDS AT THE NEW HOSPITALS.

The South London Hospital for Women has done wisely in attracting to itself the interest of various lay bodies of workers; and as an instance of what can be done on such lines we may mention the fête which was organised the other day by the Teachers' League connected with the hospital. The aim of the League is to assist the hospital, if possible, by the endowment of a bed in a private ward for members of the teaching profession who cannot afford to pay nursing-home fees, and the fête was held partly to raise funds for this object and partly for the benefit of the general funds of the hospital. Miss Chadburn, M.D., the president, in a brief speech, drew attention to the need for medical women, especially in India, and to the hospital's work in training them. It is interesting to note how much effort is at last beginning to be aroused on behalf of pay-wards, and we may hope to find before very long, if the movement grows as it promises, that a hospital unprovided in this respect will be an exception rather than the rule. The benefits arising under the classified wards of American hospitals will thus be forthcoming in some degree in this country.

### ASYLUMS BOARD STAFF AND UNION BADGES.

After some discussion the Managers of the Metropolitan Asylums Board have decided to permit members of their staff to wear the badges of the unions or organisations to which they belong. Applications for permission to do so have been received from various branches of the staff, and eventually the permission recommended by the General Purposes Committee was agreed to. In the course of the discussion, however, Mr. H. Mount Sowerby declared that permission to wear badges would only lead to coercion. What, were it given, he asked, was to prevent nurses who belonged to Suffragist organisations from wearing badges? Whereupon Mr. Highley asked why Suffragist nurses should be prevented from wearing badges in the Board's hospitals? Fortunately this attempt to draw a red-herring across the debate was defeated by Mr. Garrity's humorous assertion that the men had as much right to wear trade-union badges as the Managers had to don Masonic emblems. The common sense of the matter appears to be that, in the light of the absence of harm resulting from the London County Council's permission to the wearing of badges by its employees, some definite proof that the practice interferes with proper discipline must be brought forward if the refusal is not to cause unnecessary exasperation.

### A NEW POOR-LAW INFIRMARY FOR DERBY.

A Sub-Committee of the Derby Guardians appointed to consider the classification of the inmates of the workhouse have decided to recommend the Board to build a new infirmary for 250 beds on a piece of land adjoining the present workhouse. In support of this proposal the Committee say that it is impossible to classify the inmates efficiently in the existing buildings; that every ward is occupied to the fullest extent; that the number of admissions, especially to the sick wards, is increasing every year; and, finally, that the removal of the sick would enable suitable provision to be made in the workhouse for epileptics and feeble-minded, of whom there are ninety-three now in the institution. There are 190 patients at present in the sick wards of the workhouse—a number quite sufficient to justify the Committee's recommendation of a separate infirmary. The cost of building an infirmary of 250 beds is estimated at £20,000; £1,000 is to be allotted for isolation blocks, and the same amount for an operating-theatre, dispensary, and surgery. These estimates are low if the proposed infirmary is to fulfil the requirements of modern hospital construction. If the Derby Guardians decide to adopt this scheme, complete administrative separation of the new infirmary should be effected.

### STAFF RESIGNATIONS AT BARRY HOSPITAL.

The voluntary staff at the Barry Accident Hospital, Glamorganshire, which consists of six medical men, have sent in their resignations to the

District Council, giving one month's notice. For some time past the staff has been of a temporary character, subject to three months' notice, in view of the hospital committee's proposal to appoint a resident medical officer at a salary of £600 a year. Councillor Murrell, chairman of the hospital committee, has expressed the opinion that the situation was foreseen, since the staff had openly declared that they would not work with any person appointed at such a salary. The committee believed other medical men were willing to do so, and it is proposed to alter the present system under which the rota is prepared by the medical men. The Barry Hospital, which was founded nineteen years ago for accidents and surgical cases, possesses twenty-six beds, and was taken over by the Barry District Urban Council from the local Nursing Association in 1900. The committee consists partly of members of the Council and partly of members of the local medical profession.

### THE PROCEEDS OF ALEXANDRA DAY.

The distribution of the proceeds of the Alexandra Day collections shows signs that the movement has improved its organisation, and reduced the administrative expenses which were once an unfortunate feature of the collection. At a meeting of the Distribution Committee, which took place on Monday at the Mansion House, with the Lord Mayor in the chair, Miss C. May Beeman, the secretary, reported that after all expenses had been paid there would be about £22,000 to distribute. This sum is £6,000 more than the available total last year, and it is interesting to note that the report also states that the chief item of expenditure in connection with the collection was for the manufacture of the actual roses which were sold. It was also reported that Queen Alexandra, at the wish of the Committee, had indicated what institutions should benefit so far as the sum of £6,645 was concerned. The remainder was allocated by the Distribution Committee at the meeting.

### SUGGESTED NEW HOSPITAL FOR BLACKPOOL.

We are glad to see that the Blackpool papers, last week, which hitherto have done little more than record the see-saw of the long dispute between the local doctors and the board of management of the Victoria Hospital, now at last have declared that "the public should demand an impartial and thorough inquiry." They proceed to add that "it behoves the Mayor to take the initiative." The occasion for this belated attempt to arouse public opinion is the suggestion that the local doctors, whose boycott of the hospital still prevails, should not be content with this negative policy (since doctors can be imported there is still a staff), but should open a cottage hospital themselves. The idea is for the doctors to raise the necessary money for the conversion of the premises in Park Road—which they would adapt for the work of a cottage hospital—and then for the public to be appealed to for maintenance and support. It was hoped some days ago that these premises, which are believed to be suitable, could be got into working order early this month, but a hitch occurred; with it there disappeared the prospect of immediate possession. Whether a new hospital would gain the support of those individual subscribers who have joined the companies which support the old we cannot say; nor is it clear whether there is room for two hospitals in Blackpool, though it is almost certainly with less hospital beds than it requires, like other places. But so long as no subscriber or group can be found with the necessary public spirit to insist on a general meeting being called and a full inquiry being undertaken, we are afraid that even a cottage hospital started by the local doctors would not lead to any ending of their boycott of the Victoria Hospital, nor do otherwise than encourage the present board still to hang on in face of professional opposition. Under the circumstances a wise board would leave it to the subscribers to decide the dispute. As we go to press another conference is being held between the two sides.

### "SECRET REMEDIES" VINDICATED.

The result of the libel action brought by Mr. Stevens, managing director of a company for the manufacture of an alleged cure for consumption, against the British Medical Association, which he contended was contained in the well-known volume "Secret Remedies: What They Cost and What They Contain," has resulted in a verdict for the defendants. Mr. Justice Shearman asked the jury to decide whether the words were libellous, and whether the authors of them went beyond fair criticism. He said that the jury would have to consider whether the plaintiff believed in the claims which he made. That, he added, might be the keynote of the whole case, since one of the charges made by the defendants was that the sellers of alleged consumption cures set out deliberately to deceive. It is hardly necessary to point out how important this verdict is, for it vindicates the methods employed in "Secret Remedies," by which the profession seeks to acquaint the public with a simple knowledge of what these patent medicines are made, and of the often trifling cost of their preparation. Had it been lost, it would no doubt have been the signal for an attack on "Secret Remedies" by many of the proprietors of the preparations analysed in it; but with the verdict in favour of this useful book, we may take it that to tell the public what a proprietary medicine is made of and how little it costs to make can never again be considered libellous.

### A SURREY SANATORIUM FOR WOMEN.

The latest addition to large sanatoria is that which is to be erected at Hyde Hill, two miles from Godalming, on a site of fifty-seven acres of land which has been purchased by the Metropolitan Asylums Board. There are to be 200 beds for women patients, and the site is 400 feet above the sea. The plans for the building are being prepared, and will be awaited with considerable interest, since the designing of sanatoria has undergone considerable modifications of recent years. First, there has been an attempt to economise the cost of building; and, secondly, now that the treatment has been altered so much from the days when rest was practically the only prescription, the attempts made to find suitable work for patients all raise problems which affect the planning in several respects.

### PANEL DOCTORS AND DISCLOSED NAMES.

The Medical Committee of the Dundee Insurance Committee recently suggested that the names of persons concerned with cases of complaint reported on by the Medical Service Sick Committee should not be printed in that Committee's reports, and that the persons concerned should be referred to only as the patient and the doctor, "or such other terms as will secure that identification is impossible." At the meeting which considered the suggestion a letter was read from Mr. A. W. Anderson, the chairman of the Medical Service Sub-Committee, in which he declared that the suggestion, in his opinion, was not only *ultra vires* but also unnecessary. So far as he knew the Commissioners' regulations, which prescribed that the proceedings of the Sub-Committee should be private, have never been violated, and that condition had been always preserved. No name either of doctor or patient had appeared in any of the Sub-Committee's reports. Dr. R. C. Buist thereupon declared that the matter was one of considerable difficulty, and that it was to be considered by the Commissioners in connection with revised regulations as to whether the publication of names should not be definitely prohibited. Publicity, he asserted, was not so much a protection as a danger, and they did not want that danger to arise. The Medical Committee's motion was eventually lost, and it is not one which is calculated to increase the confidence of local insured persons in their panel doctors.

### A LONG CASUALTY LIST.

The death on July 26 of Dr. G. Allpress Simmons, senior radiographer to St. George's Hospital, and formerly in charge of the same department at St. Mary's, adds one more to the extraordinary list of casualties among the St. George's staff during the last ten years. During that period Mr. Dent, senior surgeon; Mr. Allingham, assistant surgeon; Dr. Lee Dickinson, assistant physician; and Dr. Simmons have died in harness. Mr. Sheild, surgeon; Mr. Jaffrey, surgeon; Mr. Jones, assistant surgeon; and Dr. Spriggs, assistant physician, have had to resign owing to ill-health; while Sir William Bennett, Sir Isambard Owen, Dr. Penrose, and Dr. Dakin have retired for various reasons before the expiration of their full terms of service. Indeed, the only members of the staff to retire on account of the age limit in the last twelve or thirteen years have been Dr. Ewart, late senior physician, and Mr. Bull, late aural surgeon. The result of these numerous casual vacancies has been to provide St. George's with a staff considerably younger, on the average, than that at most other London hospitals. Not that this is any disadvantage—indeed, probably the reverse in regard to professional efficiency; though from the standpoint of administration and policy it may be partly accountable for recent happenings there.

### A HOSPITAL PRESIDENT'S WARNING.

Addressing his tenants at Lydney, Gloucestershire, recently, Mr. Charles Bathurst voiced a serious implied criticism on the local hospital. The institution, he said, had yet to satisfy those who naturally looked to it to provide treatment for serious accidents. They must see that it did provide for their needs in these emergencies. The medical men, he went on, whatever their difficulties, had got to see that serious cases of possibly fatal accidents were treated at once on the spot, and not sent away to Gloucester. If such cases—and the recent one was unfortunately not the first—were sent away in this manner, he would have to say that the hospital was not fulfilling the purpose for which it was founded. It is indeed time that an end was put to the difference which has arisen between the doctors on the one hand and the local industrial community on the other. Coming as they do from the President, these words should be sufficient to show both parties that an agreement must be come to without further delay.

### THE LONDON AMBULANCE SERVICE.

It now seems highly probable that the London County Council's Ambulance Service will be in operation by the end of the year. Nine motor ambulances have already been ordered, and the construction of them is being proceeded with rapidly. It is hoped that the delivery of the first ambulance will be made in October. Six ambulance stations have been selected, these being at North End Road, Fulham; Herbrand Street, Euston Road; the Boundary Street Estate, Shoreditch; the Elephant and Castle, Newington; High Road, Lee; and Brixton Hill. In the six stations accommodation will be provided for seven ambulances, which will not necessarily be in active service simultaneously. By the purchase of nine ambulances it is felt that an adequate margin for disablement and repairs is provided. In five cases new buildings will be required, and it is suggested that the ambulance station should be a building of wood covered with galvanised corrugated iron and lined with asbestos sheeting. The central office of the Ambulance Service will be at the chief station of the Fire Brigade. It is proposed to order immediately three thousand tablets with a letter "A" in red on a white ground, which are to be fixed to buildings, the owners or occupiers of which will allow police officers access to the telephone for the purpose of summoning the ambulance.

### THE HOME FOR EPILEPTICS, MAGHULL.

An interesting account of work and a surprising amount of developments were revealed at the Home for Epileptics, Maghull, on the recent visit of the Lord Mayor of Liverpool. The number of patients has been 323, which is roughly divided in equal proportions between the sexes. The main event of the past year has been the opening of the Sir Alfred Jones Home on the Chapel House estate, with the consequent transfer to that institution of the third-class female patients at the Manor House, which was thereupon converted into a home for second-class male patients. Owing to the Mental Deficiency Act the committee had been notified that no patients who were feeble-minded could be retained in the home. Those responsible, according to that Act, for such patients had been asked to remove them, but the committee did this reluctantly owing to their experience that their arrangements for the care of sane epileptics were also valuable for those threatened with feeble-mindedness. Agricultural and horticultural occupations were found for the men, and household and laundry work for the women; under the engineer the more intelligent also carried out repairs. Recreations and sports are much in vogue, and tennis and games clubs generally run with marked administrative and financial ability. Next year a further development is taking place with the opening of an up-to-date building for men of the working class, for whom, it appears, provision is very badly needed. The new home comes into existence as the result of the generosity of the late Mr. Thomas Bartlett, who bequeathed a sum of £20,000, which is to be expended upon it. The developments here on systematic and carefully-thought-out lines are such as Liverpool may be proud of, and it is to be hoped that those responsible for the working of the Mental Deficiency Act will be careful to profit by the experience which has been gained in institutions of this class.

### THE ISOLATION PROBLEM AT BRISTOL.

Dr. D. S. Danes, Medical Officer of Health for Bristol, draws attention in his report for 1913 to the scarcity of isolation hospital accommodation in his district. For this reason he confesses that very rigid selection of cases had to be made during the year, and that there have been at times as many as one hundred applications for removal on the urgent list and not a vacant bed at the hospitals. At ordinary times, he explains, selection usually ensures the removal of such cases where home isolation is quite impossible; but under the circumstances obtaining during this outbreak very many cases were left at home under conditions which ensured the spread of infection. The pressure of scarlet fever had also the effect of immediately disturbing the arrangements made for accommodating phthisis patients at Ham Green Hospital and of limiting the accommodation for diphtheria. Serious trouble would have arisen had diphtheria been prevalent while the accommodation was thus overtaxed by scarlet fever patients. In short, accommodation provided only for less than one-half of the total cases recommended for hospital treatment by the medical attendant. The maximum number of beds available for scarlet fever during this period was 172. If the Local Government Board's Regulations were observed—viz., one bed per 1,000 population—363½ beds would be provided, whereas at present the proportion of beds per 1,000 population is 0.4. Deaths, fortunately, were very few; but an epidemic of the kind referred to once more raises the problem of whether isolation hospital accommodation must always be provided on a scale needed should the spasmodic emergency of an epidemic occur. The matter has never yet been practically decided any more than the kindred problem of what should be done with smallpox hospital accommodation when there are no cases for a long period.

### A COTTAGE HOSPITAL FOR HARWICH.

Some time ago a cottage hospital was mooted for Harwich and Dovercourt, and as the need for such an institution had often been insisted on, collections were made, and the nucleus of a hospital fund was started. Various meetings have since been held, which culminated in an announcement made by Colonel A. J. H. Ward that the trustees of the late Mr. Whitaker had offered £1,000 towards the provision of a cottage hospital, with the further sum of £5,000 for endowment purposes. This brought the building fund up to £1,191; annual subscriptions have reached £20 10s.; there is, of course, the endowment fund referred to, and this result has been achieved without, as yet, any appeal to the public. It has thereupon been decided to proceed with the scheme. Trustees have been chosen, and Mr. S. Vinnell has been appointed secretary, and Major Nalbrough honorary treasurer. Mr. Vinnell has been instructed to make inquiries in the neighbourhood as to what properties exist capable of adaptation to hospital purposes, and Lord Claud Hamilton has been appointed President. With the above financial send-off it is much to be hoped that an appeal will be issued and meet with sufficient success to justify the committee in building a new cottage hospital, as adapted premises are always costly, and neither economical nor satisfactory in the long run.

### THIS WEEK'S DRUG MARKET.

Business has been very quiet this week, and the Drug Market, like other markets, has experienced the disturbing influences of the crisis in the Near East and the unsettled political situation in Ireland. Both matters have formed the chief topic of conversation in drug-trade circles and have served to divert attention from business. Few noteworthy changes in prices have occurred during the week. The strong position of opium is well maintained, and the general opinion is that lower prices are not to be expected in the immediate future; morphine has an advancing tendency in price in sympathy with the raw material. Camphor is quiet and unchanged. There is no alteration in the quotations for mercurials, and the position of quicksilver is still unsettled. Thymol is substantially dearer. Cod-liver oil is extremely dull and quotations are nominally unchanged. The price of ergot of rye tends upwards and that of essence of lemon has a lower tendency. Orris root is lower in price. At the recent public auction of drugs in Mincing Lane Cape aloes was somewhat cheaper for better qualities; Carthagena ipecacuanha was substantially lower; jalap had a lower tendency in price; grey Jamaica sarsaparilla was a trifle dearer.

